# Evaluating the Immunogenicity of a Recombinant *Bacillus subtilis* Expressing LTB-Fused Protective Antigen of Transmissible Gastroenteritis Virus in a Murine Model

**DOI:** 10.3390/biology15020116

**Published:** 2026-01-07

**Authors:** Rongxing Fan, Yuanqi Bi, Shanshan Yang, Shaopeng Yao, Wen An, Zhongtian Wang, Zengjun Ma, Ping Rui, Tao Song, Lili Wang, Fengsai Li

**Affiliations:** 1 College of Animal Science and Technology, Hebei Normal University of Science and Technology, Qinhuangdao 066000, China; 15736758989@139.com (R.F.); 13904461105@163.com (Y.B.); shanshan0321@163.com (S.Y.); y17332956884@163.com (S.Y.); 18330396617@139.com (W.A.); 15133833105@163.com (Z.W.); mzj0712@hevttc.edu.cn (Z.M.); rp1969@126.com (P.R.); songtaoer@126.com (T.S.); iamwanglili100@163.com (L.W.); 2 Hebei Key Laboratory of Veterinary Preventive Medicine, College of Animal Science and Technology, Hebei Normal University of Science and Technology, Qinhuangdao 066000, China

**Keywords:** transmissible gastroenteritis, *Bacillus subtilis*, AD antigenic epitope, the B subunit of the heat-labile enterotoxin, immunogenicity

## Abstract

Transmissible gastroenteritis (TGE), caused by the TGE virus (TGEV), is a highly contagious enteric disease characterized by vomiting, dehydration, and watery diarrhea. It mainly endangers piglets within two weeks of age, with a 100% mortality rate, inflicting severe economic losses on the global swine industry. Since enteric tropism of the virus and mucosa serves as the first line of defense against viral invasion, an oral vaccine inducing sufficient secretory immunoglobulin A (SIgA) antibodies in animals should be developed. Being a generally recognized as safe (GRAS) microorganism, *Bacillus subtilis* can form endospores under extreme environmental conditions, which confer resistance to the hostile gastric environment and have been widely employed as delivery vehicles for oral vaccines owing to their immunoadjuvant activity and non-specific antidiarrheal effects. In this study, the AD antigenic epitope of the TGEV S protein was selected as the immunogen. The mature peptide of the B subunit of the heat-labile enterotoxin from enterotoxigenic *Escherichia coli* served as a mucosal adjuvant, and *B. subtilis WB800N* was used as the delivery host to construct the recombinant strain pHT43-LTB-AD/*WB800N*. After confirming the successful expression of the target protein, oral immunization was performed using mice as a model. The results demonstrated that this recombinant strain induced robust mucosal, humoral, and cellular immunity, along with considerable levels of neutralizing antibodies. These findings indicate that recombinant *B. subtilis* could serve as an oral vaccine candidate to combat TGEV infections.

## Simple Summary

Transmissible gastroenteritis is a severe intestinal disease that causes high mortality in newborn piglets, leading to major economic losses in the swine industry worldwide. Since the pathogen infection starts in the gut, it is crucial to develop an oral vaccine that could trigger strong immune responses in the intestinal mucosa. In the study, a food-grade bacterium, *Bacillus subtilis*, was used to deliver a key viral protein fused with an immune-boosting component to create a safe oral vaccine candidate. After constructing the recombinant strain and confirming protein expression, we administered it orally to mice. The results showed that this candidate successfully stimulated comprehensive immune protection, including gut immunity, antibody production, and cellular responses, while also generating effective virus-neutralizing antibodies. Our findings demonstrate that this *Bacillus subtilis*-based oral vaccine candidate is a promising candidate for controlling transmissible gastroenteritis, offering a practical and effective solution to protect piglets and reduce economic impacts on the swine industry.

## 1. Introduction

Transmissible gastroenteritis virus (TGEV) causes a highly contagious intestinal disease in pigs, characterized by vomiting, dehydration, and profuse watery diarrhea [1]. The infection is most severe in neonatal piglets under two weeks of age, with up to 100% mortality rate owing to their underdeveloped digestive systems and immature immunity, causing substantial economic loss to the swine industry [2]. Since its discovery in the United States of America in 1945 and subsequently in other countries, the virus was first isolated in China in 1973 and has since become endemic to many provinces. The recent fluctuating infection rates observed across various regions underscore its ongoing threat as a significant pathogen in the Chinese swine industry [3]. TGEV is an enveloped and pleomorphic RNA virus with approximately 28.5 kb genome encoding four structural proteins [4]. The spike (S) protein is a structural membrane protein that mediates host cell binding and neutralizing antibody elicitation and is consequently regarded as a prime target for vaccine design [5]. It is processed into S1 and S2 subunits, with the globular S1 subunit mediating both host cell receptor binding and neutralizing antibody elicitation [6]. In previous studies, it has been established that the S1 protein contains four major antigenic sites, designated as C, B, D, and A. Among the four known antigenic sites, the surface-exposed site A and highly conserved site D are pivotal epitopes that induce this protective immune response. Site A comprises three subsites (Aa, Ab, Ac) with key residues at positions 538, 591, and 543, respectively, whereas site D is located at residues 382–385 [7]. Owing to its critical role, the AD antigenic site in the S1 subunit is a highly attractive target for vaccine development.

Traditionally, inactivated and attenuated vaccines have been the primary treatment modalities developed for TGEV [8]. However, these platforms often fail to elicit robust mucosal IgA responses (inactivated vaccines) and carry the risk of virulence reversion (attenuated vaccines) [9,10]. Owing to the enterotropism and fecal–oral transmission route of TGEV [11], developing an effective oral vaccine is crucial for inducing protective mucosal immunity and controlling disease spread. *Bacillus subtilis* is a non-pathogenic Gram-positive bacterium with generally recognized as safe (GRAS) status [12]. Its robust spore form confers high resistance to gastrointestinal stressors, such as acid and bile, enabling effective oral delivery and stabilization of recombinant antigens [13]. Furthermore, *B. subtilis* not only functions as a mucosal antigen carrier but also exhibits intrinsic immunoadjuvant properties by activating antigen-presenting cells and promoting cytokine release [14]. Combined with its probiotic ability to modulate gut microbiota and enhance host immunity [15], -with studies demonstrating its efficacy in inhibiting TGEV entry into intestinal epithelial cells [16], these attributes collectively establish *B. subtilis* as a versatile and potent vehicle for mucosal immunization and oral biological delivery. Mou et al. have demonstrated that an oral vaccine based on recombinant *B. subtilis* spores delivering the TGEV S protein could effectively induce mucosal SIgA and systemic IgG responses by targeting intestinal dendritic cells and promoting their migration to mesenteric lymph nodes, thereby laying a mechanistic foundation for the development of next-generation TGE vaccines based on mucosal immunity [17].

Heat-labile enterotoxin (LT), an exotoxin produced by enterotoxigenic *Escherichia coli* (*ETEC*), comprises an A subunit (LTA) and a non-toxic B subunit (LTB) [18]. LTB functions as a potent mucosal immunoadjuvant, eliciting strong immune responses by modulating Th1/Th2-type cytokine profiles [19]. It also disrupts immune tolerance, thereby facilitating long-lasting immunological memory against associated antigens [20]. Lei et al. demonstrated that intranasal delivery of an LTB–H5N1 recombinant vaccine in chickens elicited robust immune responses with elevated IgG, secretory Immunoglobulin A (sIgA), and neutralizing antibodies while providing complete protection, supporting its development as a mucosal vaccine candidate [21]. Jiang et al. demonstrated that oral immunization with *Lactobacillus plantarum* co-expressing recombinant HA2 and LTB proteins elicited protective immunity in mice, significantly reducing mortality upon challenge with avian influenza virus [22]. Taken together, these findings provide strong evidence supporting the role of LTB as a potent mucosal immunoadjuvant.

In this study, recombinant *B. subtilis* pHT43-LTB-AD/*WB800N* was engineered using *E. coli–B. subtilis* shuttle plasmid, pHT43-His, to co-express the antigenic AD domain of the TGEV S protein as the target immunogen and LTB as a mucosal adjuvant. In a murine oral immunization model, the immunogenicity of the recombinant strain pHT43-LTB-AD/*WB800N* was comprehensively assessed by examining mucosal, humoral, and cellular immunity, with neutralizing antibody titers serving as key correlates of protection.

## 2. Materials and Methods

### 2.1. Virus, Bacterium, Plasmids, and Cell Line

The strain TGEV HB-1 (GenBank accession no.MZ368889) was isolated from virus-positive samples collected from pig farms during severe outbreaks of acute diarrhea in piglets. *B. subtilis WB800N* and plasmid pHT43-his were acquired from Wuhan MiaoLing Biotechnology Co., Ltd (Wuhan, China). Swine testicular (ST) cells from our laboratory repository were used for TGEV propagation and cultured in Dulbecco’s modified Eagle’s medium (DMEM; Gibco, Carlsbad, CA, USA) supplemented with 10% fetal bovine serum (FBS; Gibco, Thermo Fisher Scientific, Waltham, MA, USA).

### 2.2. Construction of Recombinant B. subtilis pHT43-LTB-AD/WB800N

**Figure 1:**
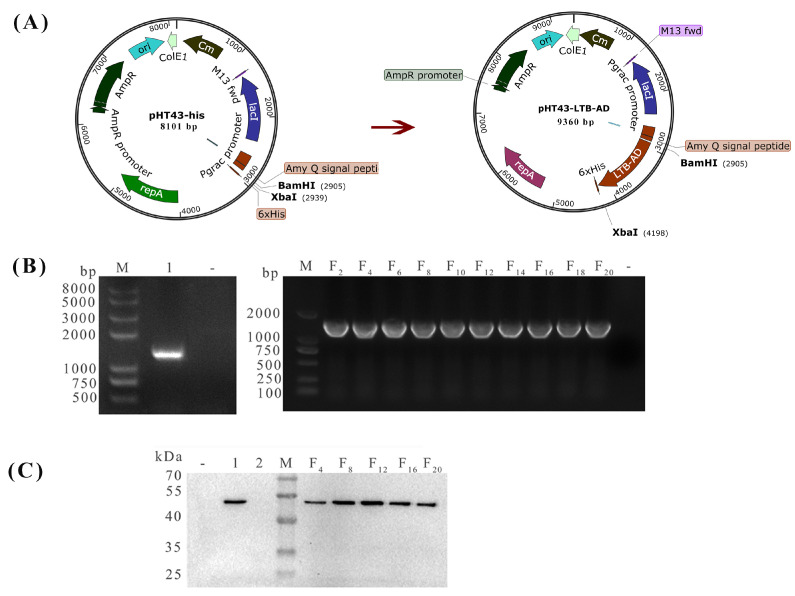
Construction and verification of the recombinant *Bacillus subtilis* pHT43-LTB-AD/*WB800N* and expression analysis of the target protein. (**A**) A schematic diagram of the construction of DNA plasmids. pHT43-his: an *E. coli*–*B. subtilis* shuttle vector featuring a strong Pgrac promoter, an α-amylase signal peptide, and a His-tag. It confers ampicillin and chloromycin resistance for selection. LTB: Mature peptide sequence of the heat-labile enterotoxin B subunit; AD: Protective antigen of transmissible gastroenteritis virus (TGEV). (**B**,**C**) Identification and analysis of the stability of *B. subtilis* strain pHT43-LTB-AD/*WB800N* with agarose gel electrophoresis and Western blot. M: Trans 2k plus II DNA marker; 1: The plasmid pHT43-LTB-AD; -: Negative control (**B left**). M: DL2000 DNA marker; F_2_–F_20_: The F_2_–F_20_ generation plasmid pHT43-LTB-AD; -: Negative control (**B right**). M: Protein molecular weight marker; 1: The strain pHT43-LTB-AD/*WB800N*; 2: The strain pHT43/*WB800N;* F_4_–F_20_: The F_4_–F_20_ generation strain pHT43-LTB-AD/*WB800N;* −: Negative control.

A schematic of the recombinant DNA plasmid construction is shown in Figure 1A. Briefly, the AD fragments of the S protein gene (the A antigenic domain, the intervening spacer region and the D antigenic domain) showing >99.9% sequence identity with contemporary circulating strains (Supplementary Figure S1) was amplified and purified from the TGEV HB-1 cDNA sequence using primers AD-F and AD-R (Table 1). The mature peptide sequence of LTB was amplified and purified from plasmid pUC57-LTB (Tsingke Biotechnology Co., Ltd., Beijing, China) using the primers LTB-F and LTB-R (Table 1). The purified PCR products LTB and AD were subsequently cloned into the vector pHT43-his at the *BamH* I and *Sma* I restriction sites to generate the plasmid pHT43-LTB-AD using a multi-fragment recombination strategy. All plasmids were identified by double enzyme digestion, PCR and sequence verification (pHT43-F/R primers; Table 1).

To construct the recombinant strain pHT43-LTB-AD/*WB800N*, *B. subtilis WB800N* competent cells were prepared as described previously [23], followed by chemical transformation [24]. The cryopreserved *WB800N* strain was streaked on a Luria–Bertani (LB) agar plate and incubated at 37 °C for 10 h. A single colony was then inoculated into SPI medium, followed by subsequent culturing in GM I and GM II media to induce competence, and the cells were aliquoted for immediate use. The recombinant plasmid pHT43-LTB-AD was added to the competent cells at a final concentration of 1 µg/mL, with the volume not exceeding 1/20 of the cell suspension, and the mixture was incubated at 37 °C for 30 min. Subsequently, the cells were cultured with shaking for 2.5 h, spread onto LB agar plates containing 10 µg/mL chloramphenicol, and incubated at 37 °C for 12–18 h. The pHT43/*WB800N* strain was prepared as previously described. The plasmid was extracted, and the presence of the sequence encoding the fusion protein LTB-AD in the recombinant plasmid was confirmed by PCR using primers pHT43-F/R.

To analyze the expression of the target protein by the recombinant strain pHT43-LTB-AD/*WB800N*, the bacteria was cultured at 37 °C in LB medium containing 10 μg/mL chloromycin with shaking. As the OD_600_ increased to 0.8, IPTG was added to a final concentration of 1 mM to induce expression at 37 °C for 24 h. Bacterial cells were harvested by centrifugation at 12,000× *g* for 10 min at 4 °C, and the pellet was disrupted by ultrasonication. The supernatant was collected, treated with 5× sodium dodecyl sulfate (SDS) for 10 min at 100 °C, and stored at −20 °C for subsequent use. *B. subtilis* pHT43/*WB800N* was subjected to the same procedure and used as a control. Equal amounts of protein samples were electrophoresed on a 12% SDS–polyacrylamide gel and subsequently transferred on 0.45 μm polyvinylidene fluoride (PVDF) membranes. The membranes were probed with a commercial mouse anti-His monoclonal antibody (1:1000 dilution) as the primary antibody, followed by horseradish peroxidase (HRP)-conjugated goat anti-mouse IgG (1:5000 dilution) as the secondary antibody. Immunoreactive bands were visualized using an enhanced chemiluminescence (ECL) substrate, according to the manufacturer’s instructions.

To assess the plasmid retention capacity of the bacterial strain pHT43-LTB-AD/WB800N, both genetic and protein expression analyses were performed. The strain was cultivated at 37 °C in LB broth containing chloramphenicol with shaking for 10 h. Plasmid DNA was extracted at every second generation and analyzed by PCR using the primers pHT43-F/R. For protein analysis, cultures were grown in chloramphenicol-supplemented LB medium with shaking to an OD_600_ of 0.8, induced with IPTG, harvested, and lysed by sonication. After centrifugation, the supernatant was collected as the protein sample every fourth generation and subjected to Western blot analysis using mouse anti-His monoclonal antibody as the primary antibody. Plasmid stability was monitored over 20 successive generations.

### 2.3. Immunization Schedule

**Figure 2:**
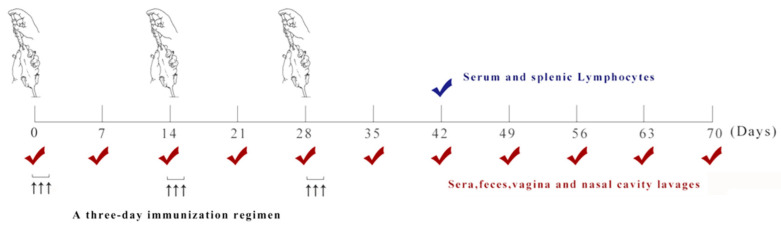
Timeline of the immunization and sample collection schedule. The immunization protocol consisted of three consecutive days of immunization (days 1–3), repeated for three cycles, followed by booster shots at 14-day intervals (days 14–16 and 28–30). The upward arrow represents the specific time of oral immunization. Serum, fecal samples, nasal washes, intestinal mucus, and vaginal lavage fluids were collected weekly and analyzed for IgG and sIgA antibody levels. On day 42, serum and splenic lymphocytes were collected in bulk for the analysis of cellular immunity.

To evaluate the immunogenicity of the recombinant strain pHT43-LTB-AD/*WB800N*, 45 4-week-old specific pathogen-free (SPF) female BALB/c mice were obtained from Shenyang Aojie Biotechnology Co., Ltd. (Shenyang, China) and acclimatized for one week under SPF conditions with free access to a standard diet and water; all procedures adhered to the relevant institutional animal care guidelines (2025025). For primary immunization, the recombinant strains were cultured in LB medium containing 10 μg/mL chloramphenicol with shaking to an OD_600_ of 0.8, at which point IPTG was added to induce expression at 37 °C for 24 h. The cells were subsequently harvested by centrifugation, washed with PBS, and resuspended to adjust the bacterial concentration to 1 × 10^10^ CFU/mL. The SPF BALB/c mice were randomly divided into three groups (*n* = 15 per group): PBS, pHT43/*WB800N*, and pHT43-LTB-AD/*WB800N*.Randomization was performed using a manual lottery procedure to ensure unbiased allocation. Briefly, each of the 45 mice was assigned a unique identifier after acclimatization. All identifiers were placed into a container, thoroughly mixed, and then drawn blindly and sequentially by an independent researcher not involved in any subsequent experimental procedures. Mice were assigned to the aforementioned groups based on the order of the draw. Following grouping, mice were immunized serially for three consecutive days at two-week intervals (days 0–2, 14–16, and 28–30). After primary immunization, serum, nasal wash, vaginal lavage, feces, and intestinal mucus samples were collected every 7 days for 70 days and the levels of IgG and secretory IgA (sIgA) antibodies were determined (Figure 2). All samples were processed according to a previously published method and stored at −20 °C until use [25].

### 2.4. IgG and sIgA Antibody Analysis

The ELISA for TGEV-specific antibodies in sample collections was conducted using TGEV-coated polystyrene microtiter plates (incubated at 4 °C for 12 h) as the solid phase, with plates coated with cultured ST cells constituting the negative antigen control. After blocking with 5% skim milk at 37 °C for 2 h and three washes with PBST, serum and mucus samples were diluted in 5% skim milk to apply to the wells and subsequently incubated at 25 °C for 2 h. After washing with PBST, 1:5000 diluted HRP-conjugated goat anti-mouse IgG/IgA antibody (Invitrogen, Thermo Fisher Scientific, Waltham, MA, USA) was added and incubated at 37 °C for 1 h. Color development was then initiated with o-phenylenediamine dihydrochloride (Sigma-Aldrich, St. Louis, MO, USA), followed by absorbance measurement at 450 nm.

Detecting neutralizing antibodies is critical for assessing immunogenicity and predicting the protective efficacy of vaccines. On day 42 post-primary immunization, mouse serum was collected to determine the neutralizing antibody capacity. Briefly, 50% tissue culture infective dose (TCID_50_) of TGEV was determined using the Reed–Muench method. Serum samples collected from immunized mice were two-fold serially diluted (total volume of 50 µL), mixed with an equal volume of TGEV (200 TCID_50_/100 µL), and incubated at 37 °C for 1 h. The treated viral preparations were then added to confluent monolayers of ST cells in 24-well plates. Following this, the cells were overlaid with 1% methylcellulose, and the plates were incubated at 37 °C in a 5% CO_2_ atmosphere. Cultures were monitored daily for five days to develop TGEV-specific cytopathic effects (CPE). The virus neutralization titer was calculated as the reciprocal of the highest serum dilution that completely inhibited CPE in 50% of the wells, using the Reed-Muench method. The geometric mean titer (GMT) for each group was then calculated from individual log-transformed titers.

### 2.5. Lymphocyte Proliferation in Response to AD Antibody Stimulation

On day 42 post-immunization, splenocytes were isolated from three mice per group and subjected to a lymphocyte proliferation assay [26]. Briefly, the cells were seeded in 96-well plates at 5 × 10^6^ cells/mL and stimulated with varying concentrations of purified AD protein (1, 5, and 25 μg/mL) for 66 h, using ConA (5 μg/mL) as a positive control. The AD protein was previously prepared and stored at −80 °C in our laboratory using the *Escherichia coli BL21(DE3)* expression system. Then, cell proliferation was assessed using a 3-(4,5-dimethylthylthiazol-2-yl)-2,5-diphenyltetrazoliumbromide (MTT) assay. After incubating with MTT for 4 h, the formazan crystals were solubilized with dimethyl sulfoxide (DMSO), and the absorbance was measured at 570 nm. The experiment was performed in triplicate and the splenocyte proliferation index (SI) was calculated as the mean optical density (OD) of the antigen-stimulated wells divided by the mean OD of the negative control wells.

### 2.6. Analysis of CD4^+^ and CD8^+^ T-Cell Subsets

On day 42 post-primary immunization, 1 × 10^6^ splenocytes/mL were analyzed by flow cytometry (FCM) to determine the proportions of CD4^+^ and CD8^+^ T lymphocytes. T cells were stained with PE-conjugated anti-mouse CD4 and APC-conjugated anti-mouse CD8 antibodies in dark at 37 °C for 30 min, washed, and resuspended in PBS (pH 7.2) for analysis using a FACSCalibur flow cytometer (BD AccuriTM C6 PLus; BD Biosciences, Franklin Lakes, NJ, USA).

### 2.7. Cytokine Detection

On day 42 post-primary immunization, serum samples were collected for cytokines analysis (IFN-γ, IL-4 and IL-17) using commercial OptEIA™ ELISA Kits (Biosource International, Camarillo, CA, USA), with concentrations calculated from a standard curve fitted by linear regression.

### 2.8. Statistical Analysis

All data were presented as mean ± standard error (SE) and analyzed using GraphPad Prism 8.0. Statistical comparisons between the treatment and control groups were performed using one-way analysis of variance (ANOVA), followed by Tukey’s post hoc test. * *p* < 0.05 and ** *p* < 0.01.

## 3. Results

### 3.1. Construction and Stability Assessment of the Recombinant B. subtilis pHT43-LTB-AD/WB800N

The strain pHT43-LTB-AD/*WB800N* was grown in LB broth containing chloramphenicol-resistant shaking for 10 h at 37 °C, and the plasmid pHT43-LTB-AD was analyzed by PCR using pHT43-F/R as the primers. A 1687 bp DNA band corresponding to the expected size was observed in pHT43-LTB-AD/*WB800N* (line 1), but not in pHT43/*WB800N* (line 2), confirming the successful cloning of the LTB-AD gene into the *B. subtilis WB800N* (Figure 1B). To further validate the successful expression of target protein, the strain pHT43-LTB-AD/*WB800N* was cultured with shaking in LB medium supplemented with chloramphenicol until reaching an OD_600_ of 0.8, induced with IPTG, harvested, lysed by sonication, and centrifuged to obtain the supernatant for Western blot analysis to validate target protein expression. The expected 47 KDa immunoblot band was present in the pHT43-LTB-AD/*WB800N* strain (line 2) but not in the pHT43/*WB800N* strain (line 3), verifying the successful expression of the LTB-AD protein in *B. subtilis WB800N* (Figure 1C).

To evaluate the plasmid retention capacity of the bacterial strain, the engineered strain pHT43-LTB-AD/*WB800N* was serially sub-cultured in LB medium for 20 generations. The plasmid pHT43-LTB-AD was examined every second generation by PCR amplification of the restriction site-flanking regions with the primers pHT43-F/R. As shown in Figure 1B, specific amplification products corresponding to the expected molecular weights were consistently detected in the pHT43-LTB-AD/*WB800N* strain, but not in the control strain pHT43/*WB800N*. In addition, the expression stability of the target protein LTB-AD was evaluated in parallel. Total protein was extracted from the serially passaged cultures (sampled every fourth generation) and analyzed by Western blot using a mouse anti-His monoclonal antibody. As shown in Figure 1C, specific immunoreactive bands corresponding to the expected molecular weights were consistently detected in the pHT43-LTB-AD/*WB800N* strain, but not in the control strain pHT43/*WB800N*. These results indicated that the target protein LTB-AD was stably maintained in the recombinant strain under the applied experimental conditions.

### 3.2. Measurement of Serum IgG and Neutralizing Antibody Levels Induced by Recombinant B. subtilis via Oral Immunization

**Figure 3:**
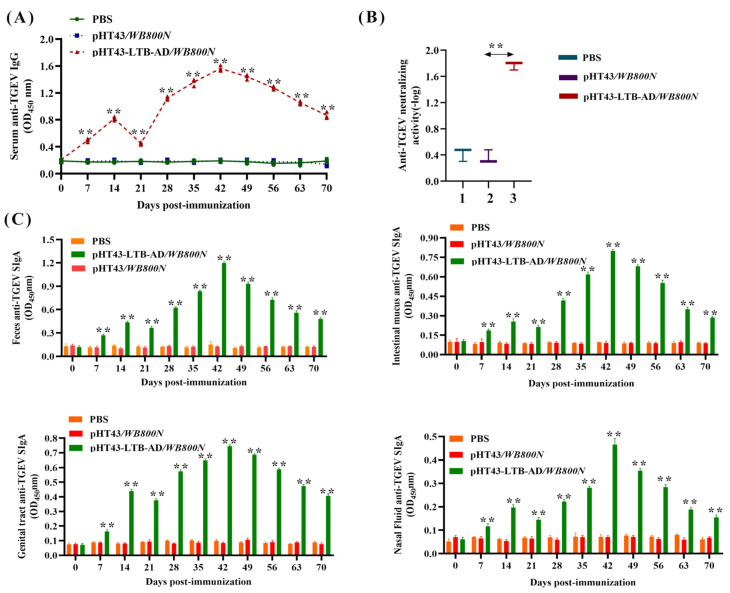
Humoral and mucosal immune responses induced by different TGEV vaccine candidate formulations were assessed by measuring serum IgG (**A**), neutralizing antibodies (**B**), and secretory IgA (sIgA) levels in feces, intestinal mucus, the genital tract, and nasal lavage fluid (**C**). Bars represent the mean SE in each group (** *p* < 0.01 compared to the control groups: pHT43/*WB800N* and PBS).

Using BALB/c mice as a model, the systemic immune response-inducing ability of pHT43-LTB-AD/*WB800N* was evaluated by measuring the levels of TGEV-specific IgG antibodies by indirect ELISA. A significant level of anti-TGEV-specific IgG antibodies was detected in mice receiving oral pHT43-LTB-AD/*WB800N* (*p* < 0.01) than in those receiving pHT43/*WB800N* and PBS from day 7 post-primary immunization, peaking at 42 days (Figure 3A). Moreover, following the booster dose, mice orally immunized with pHT43-LTB-AD/*WB800N* showed significantly higher levels of anti-TGEV IgG antibodies than those receiving pHT43/*WB800N* (*p* < 0.01). The IgG antibody levels did not change in the control group after immunization. These results demonstrated that the engineered strain pHT43-LTB-AD/*WB800N* could elicit a significant humoral immune response.

Furthermore, the anti-TGEV-neutralizing activity of serum antibodies elicited by vaccination was evaluated in mice using a fixed virus-diluted serum assay. Mice orally administered with the engineered bacterium pHT43-LTB-AD/*WB800N* developed a significantly higher level of neutralizing antibodies (1:64) than the pHT43/*WB800N* control group (1:3; Figure 3B). These results demonstrate that the TGEV AD antigen expressed by *B. subtilis* can effectively elicit a robust anti-TGEV immune response in mice.

### 3.3. Evaluation of sIgA Antibody Levels in Mice in Response to Oral Administration of Recombinant Bacteria

To evaluate the mucosal immune response induced by oral administration of strain pHT43-LTB-AD/*WB800N* in mice, the levels of specific anti-TGEV sIgA antibodies in nasal washes, feces, genital tract flushes, and intestinal mucus from immunized mice were assayed by ELISA. Mucosal sIgA levels time-dependently increased in nasal washes, feces, intestinal mucus, and genital tract flushes of mice orally administered pHT43-LTB-AD/*WB800N* compared to those of the pHT43/*WB800N*-administered mice (Figure 3C). sIgA levels began to increase from day 7 post-immunization, peaked on day 42, and subsequently declined. Notably, each booster immunization was followed by a marked increase in antibody titers. No significant differences in sIgA antibody levels were found between the control groups pHT43/*WB800N* and PBS post-immunization. These findings indicate that recombinant *B. subtilis* effectively induces a mucosal immune response in mice.

### 3.4. Lymphocyte Proliferation and CD4^+^/CD8^+^ Th Cell Analysis

**Figure 4:**
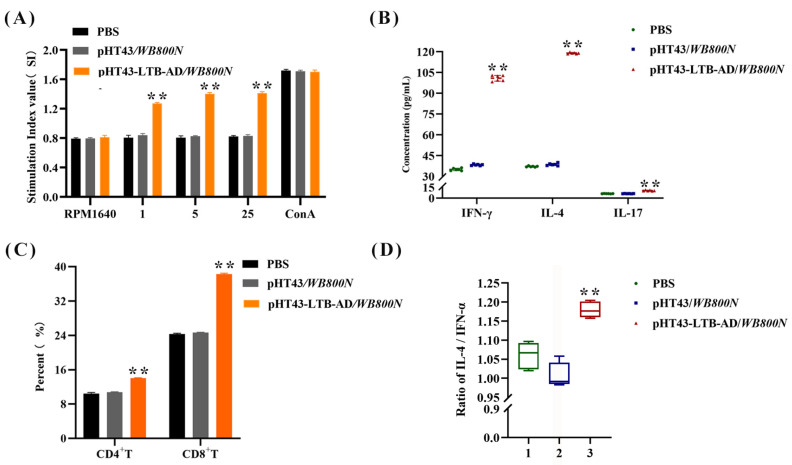
Cell-mediated immune responses induced by different vaccine candidate formulations were evaluated by assessing splenocyte proliferation (**A**), cytokine production (**B**), T-cell subpopulations (**C**), and the IL-4/IFN-γ ratio (**D**). Bars represent the mean SE in each group (** *p* < 0.01 compared to the control groups: pHT43/*WB800N* and PBS).

Splenic lymphocytes isolated from immunized mice were assessed for proliferation by MTT assay following in vitro stimulation with purified AD protein, using ConA and RPMI 1640 media as positive and negative controls, respectively. The mice immunized with strain pHT43-LTB-AD/*WB800N* exhibited a significantly higher splenocyte stimulation index than those orally immunized with strain pHT43/*WB800N* (*p* < 0.01), with the peak proliferative response observed at 5 μg/mL AD protein concentration (Figure 4A). In contrast, only a weak proliferative induction was detected in the control groups immunized with pHT43/WB800N or PBS. These results indicated that immunization with the genetically engineered strain pHT43-LTB-AD/*WB800N* elicited a robust splenocyte proliferative response specific to the TGEV AD antigen.

Moreover, to assess the populations of CD4^+^ and CD8^+^ Th cells induced by oral immunization with strain pHT43-LTB-AD/*WB800N*, splenocytes were aseptically isolated and analyzed using a FACSCalibur flow cytometer. Cells were stained with anti-mouse CD4-PE and anti-mouse CD8-APC monoclonal antibodies. Oral immunization with the recombinant strain pHT43-LTB-AD/*WB800N* significantly increased the population of CD4^+^ and CD8^+^ T cells than with the controls, with no significant difference observed between the pHT43/*WB800N* and PBS groups (Figure 4C). Collectively, these findings demonstrate that the enhanced cellular immune response was specifically mediated by the LTB-AD antigen.

### 3.5. Cytokine Analysis

Serum was collected aseptically from mice, and the IFN-γ, IL-4, and IL-17 concentrations were determined using a specific Invitrogen cytokine assay kit. The oral administration of strain pHT43-LTB-AD/*WB800N* significantly increased the IFN-γ, IL-4, and IL-17 levels than that of PBS control and strain pHT43/*WB800N* (Figure 4B,D). This suggests successful induction of Th1, Th2, and Th17 cell responses. Furthermore, the IL-4/IFN-γ ratio (an indicator of the Th2/Th1 bias) in the pHT43-LTB-AD/*WB800N* group was higher than in the other two groups. Collectively, these results indicate that the cellular immune response elicited by oral pHT43-LTB-AD/WB800N was predominantly a Th2-type response.

## 4. Discussion

Despite intense research and control efforts, TGEV poses a substantial threat to pig farming, particularly owing to its effects on young animals with immature immune systems [27]. This persistent challenge is largely attributed to the high mutation rate and efficient fecal–oral transmission of the virus, which complicates the development of broadly effective and durable vaccines [28]. Therefore, to ensure the field relevance of our vaccine development efforts, we used the TGEV HB-1 strain isolated from a porcine acute diarrhea outbreak in Qinhuangdao, Hebei Province, China and classified as belonging to the Purdue cluster based on whole-genome sequencing and phylogenetic analysis. The S protein of TGEV is the major inducer of neutralizing antibodies, as it mediates viral entry and contains critical neutralizing domains [29]. Notably, antigenic site A and the highly conserved site D (collectively termed AD) on the S1 subunit have been identified as pivotal epitopes capable of eliciting a potent protective immune response [30,31]. To ensure broad applicability, an evaluation of epitope conservation across major TGEV genotypes was conducted. Comparative sequence analysis revealed >99.9% nucleotide sequence identity between the AD domain of the HB-1 strain and contemporary circulating strains in China. Based on this rationale, the AD epitope from the S protein of the TGEV HB-1 strain was selected and amplified as a key immunogen in vaccine development.

The mucosal immune system, in which sIgA plays a predominant role, serves as the first line of defense against enteric pathogens [32]. Therefore, an oral vaccine that elicits high levels of mucosal immunity and protection against enterotropic TGEV should be developed. The *B. subtilis WB800N* utilized in this study was selected as an antigen delivery vehicle because of its tolerance to harsh gastric environments, non-specific antidiarrheal activity, and immunoadjuvant properties, making it suitable for use as a delivery system in mucosal-oriented vaccines [33,34]. To address the need for improved antigen delivery efficiency and enhanced sIgA antibody production by oral vaccines, incorporating the mucosal adjuvant LTB has been proposed as a strategic approach to induce potent mucosal immune responses [35,36]. Previous studies successfully used LTB as a mucosal adjuvant in various vaccines. The gut-targeted LTB-adjuvanted rabies VLP microparticle vaccine effectively induced comprehensive systemic, cellular, and mucosal immunity in mice, highlighting its promise as an oral rabies vaccine [37]. A recombinant *Lactobacillus casei* strain expressing the PCV2 Cap–LTB fusion antigen was successfully constructed, which elicited robust systemic and mucosal immune responses in mice upon oral immunization [38].

The robust mucosal immunity elicited by LTB fusion antigens, particularly through recombinant probiotic delivery systems, underscores the need to evaluate sIgA as a cornerstone of mucosal protection [39]. In this study, the oral administration of pHT43-LTB-AD/*WB800B* induced a potent and sustained anti-TGEV sIgA response in mice, markedly increasing immunoreactivity after the booster dose. This antigen-specific sIgA response is biologically significant, as it directly impedes viral attachment and entry into intestinal epithelial cells, thereby establishing the first line of defense against TGEV invasion [40]. Furthermore, the persistence of these sIgA antibodies suggests the potential for sustained mucosal protection, which is particularly critical for preventing clinical diarrhea in neonatal and weaned piglets, who are the most susceptible to TGEV [41]. Although sIgA serves as the principal humoral defense mechanism at mucosal surfaces, serum-derived IgG plays a substantial complementary role by diminishing the capacity of pathogens to traverse the intestinal barrier and curbing the systemic dissemination of invasive pathogens, thereby augmenting mucosal immunity mediated by sIgA [42]. Serum analysis revealed that mice immunized with pHT43-LTB-AD/*WB800B* exhibited significantly higher levels of TGEV-specific IgG antibodies than the control mice. Notably, IgG titers pronouncedly and sustainedly increased after booster immunization, indicating successful initiation of a T-cell-dependent immune response and systemic immunological memory establishment [43]. This finding is consistent with the known role of LTB as a potent immunomodulator that enhances systemic antibody responses after mucosal delivery [22]. Consistent with the findings of Mou et al., our epitope-focused vaccine also elicited robust mucosal and systemic immune responses, even when presenting a minimal antigenic determinant [17]. Beyond binding antibody titers, the critical functional question is whether these systemic antibodies possess a neutralizing capacity. A virus neutralization test was performed to assess the ability of serum IgG to inhibit TGEV infection in vitro. Sera from the pHT43-LTB-AD/*WB800B*-immunized mice showed significantly higher neutralizing antibody titers, effectively preventing viral CPE in susceptible cells. This confirms that the systemic IgG response induced by the oral vaccine candidate is both quantitative and qualitative, capable of recognizing conformational epitopes on the viral surface and disrupting the infectivity of TGEV [44]. The synergy between mucosal sIgA, which blocks viral entry into the intestinal lining, and systemic neutralizing IgG, which limits viral spread, likely constitutes the key mechanism for the comprehensive protection afforded by our vaccine candidate.

The efficacy of a vaccine is critically dependent on robust cellular immune response activation, wherein T lymphocyte proliferation, differentiation into specific subsets, and the ensuing cytokine milieu are pivotal for viral clearance and protective immunity establishment [45]. In this context, the enhanced splenocyte proliferation upon re-stimulation with the antigen serves as a primary and fundamental indicator of successful T-cell activation. Our results demonstrated a characteristic dose-dependent response, where the proliferation index initially increased with the antigen concentration, peaking at 5 μg/mL, before declining at higher concentrations. This bell-shaped curve is a well-documented phenomenon in immunology and typically attributed to the induction of high-dose tolerance or activation-induced cell death (AICD) at excess antigen levels, which can lead to T-cell exhaustion or apoptosis [46]. Nevertheless, the significant proliferative response observed at optimal concentrations unequivocally indicates the successful priming of a substantial pool of antigen-specific T cells and the generation of potent cellular immunity, thereby underscoring the immunogenicity of the recombinant *B. subtilis* vaccine candidate.

Analysis of cytokine secretion serves as a critical approach for assessing a vaccine’s capacity to induce Th1- or Th2-type immune responses, which are closely related to cellular and humoral immunity, respectively. Cytokines synthesized by CD4^+^ Th cells regulate immune system functions, including antibody production and cellular immune responses. The IL-4/IFN-γ ratio is employed to determine the Th1 or Th2 skewing of the induced immune response [47]. In this study, our analysis of cytokine levels in immunized mice revealed that oral delivery of pHT43-LTB-AD/*WB800N* significantly promoted both IFN-γ and IL-4 production. The resulting IL-4/IFN-γ ratio of greater than 1 suggests the induction of a mixed Th1/Th2 immune response with a Th2-skewed bias, as reported earlier [48,49]. Recent studies have established that inducing sIgAs against microbes and foreign immunogens constitutes a T-cell-dependent immune process [50]. In this study, the LTB-AD fusion protein delivered by *B. subtilis* effectively stimulated a mixed Th1/Th2 immune response, demonstrating the crucial role of T-cell-mediated immunity in sIgA induction. To corroborate these findings at the cellular level, we assessed T-cell-mediated immunity. Our data showed that immunization significantly expanded both CD4^+^ and CD8^+^ T-cell populations. The increase in CD4^+^ T cells provides a direct cellular basis for the observed mixed Th1/Th2 response and the subsequent T-cell-dependent sIgA induction. In parallel, an increase in CD8^+^ T cells signifies the generation of a potent cytotoxic T-cell response that plays a critical role in combating intracellular pathogens. Thus, the coordinated CD4^+^ and CD8^+^ T-cell activation underscores the ability of our vaccine candidate to induce comprehensive adaptive immunity.

The promising immunological data presented in this study should be interpreted in light of a key methodological constraint: all evaluations were conducted in a murine model rather than in the target species, pigs. This experimental design reflects the consensus that murine systems offer a standardized, cost-effective, and controllable platform for proof-of-concept and preliminary immunogenicity assessment during early-phase vaccine development. Moreover, this approach aligns with ethical frameworks for exploratory investigation and remains logistically practical before advancing to large-scale, resource-demanding studies in swine. Future studies will evaluate the immunogenicity and protective efficacy of this vaccine candidate in pigs. Animals will be orally immunized following a similar regimen, and the induction of TGEV-specific systemic and mucosal antibody responses as well as T-cell immunity will be thoroughly assessed. Finally, challenge experiments using a virulent TGEV strain should be conducted to determine the protective capacity of the vaccine candidate.

## 5. Conclusions

Oral immunization with recombinant *B. subtilis* expressing the LTB-fused TGEV AD epitope induced a comprehensive adaptive immune response in mice. The vaccine elicited a mixed Th1/Th2 response with a Th2 bias, enhanced CD4^+^/CD8^+^ T-cell proliferation and demonstrated T-cell-dependent sIgA induction. These findings establish the ability of the vaccine to concurrently activate humoral and cellular immunity, supporting its potential as an effective oral vaccine candidate.

## Figures and Tables

**Table 1:** The sequence of primers.

Target	Primer Sequences	Primers Sequences
AD	AD-F AD-R	**ggcaatcagtatgaaaaac**CTTAATTTTACTACAAATGTACAATCAGGTAa**tgatgatggtgatg***cccggg*ATCAGACGGTACACCCACTATGTTG
LTB	LTB-F LTB-R	**aaaacatcagccgta***ggatcc*GCTCCCCAGACTATTACAGAACTATG **g**GTTTTTCATACTGATTGCCGCA
pHT43	pHT43-F pHT43-R	ATTCAAAAACGAAAGCGGAC CCATTTGTTCCAGGTAAGGTAT

Restriction enzyme recognition sites applied for cloning are shown in Italic. Homologous sequence was shown in bold.

## Data Availability

The data that support the findings of this study are available from the corresponding author, Fengsai Li, upon reasonable request.

## Supplementary Materials

The following supporting information can be downloaded at: https://www.mdpi.com/article/10.3390/biology15020116/s1, Figure S1: Multiple sequence alignment of the AD domain-coding region from representative TGEV strains; Figure S2: Identification and analysis of the stability of *B. subtilis* pHT43-LTB-AD/*WB800N* with western blot. M: Protein molecular weight marker; 1: The strain pHT43-LTB-AD/*WB800N*; 2: The strain pHT43/*WB800N*; F_4_–F_20_: The F_4_–F_20_ generation strain pHT43-LTB-AD/*WB800N*; −: Negative control.
