# Supplementary material for: Evaluating the Immunogenicity of a Recombinant Bacillus subtilis Expressing LTB-Fused Protective Antigen of Transmissible Gastroenteritis Virus in a Murine Model
Source: Biology (Basel). 2026 Jan 7;15(2):116. doi: 10.3390/biology15020116 (PMC12838399; doi:10.3390/biology15020116)
Supplement: Supplementary file 1 [file biology-15-00116-s001.zip › biology-4043103-supplementary.pdf]

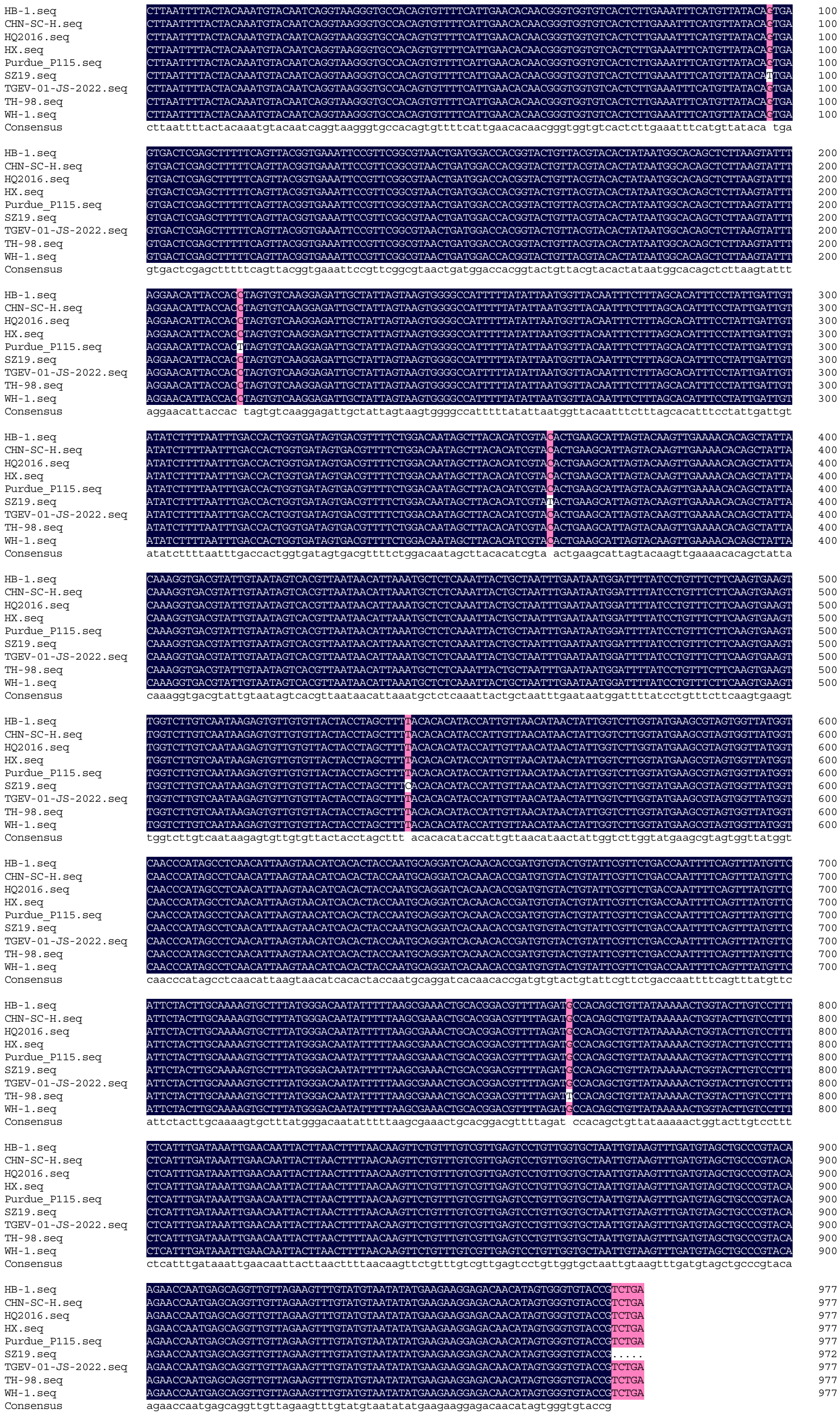

Fig. S1 Multiple sequence alignment of the AD domain-coding region from representative TGEV strains

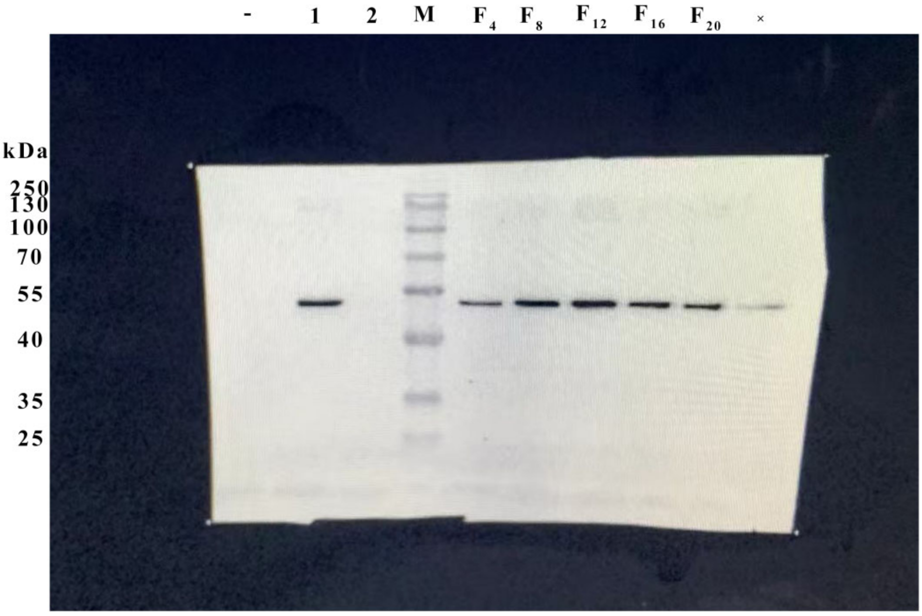

Figure S2. Identification and analysis of the stability of *B. subtilis* pHT43-LTB-AD/*WB800N* with western blot. M: Protein molecular weight marker; 1: The strain pHT43-LTB-AD/*WB800N*; 2*:* The strain pHT43/*WB800N;* F_4-F_{20}: The F_4-F_{20} generation strain pHT43-LTB-AD/*WB800N;* −: Negative control.
